# On Injuries of the Head Affecting the Brain

**Published:** 1843-04-01

**Authors:** 


### Medico-Chirurgical Review,

No. LXXVI.

[No. 36 of a Decennial Series.]

JANUARY 1, TO APRIL 1, 1843.

### On Injuries of the Head affecting the Brain. By *G. J. Guthrie*, F.R.S. Surgeon to the Westminster Hospital, and to the Royal Westminster Ophthalmic Hospital, &c. &c. 4to. pp. 155. London, 1842.

These observations are the substance of the Lectures delivered in the Theatre of the Royal College of Surgeons in the Spring of 1841. They comprise and present the results of Mr. Guthrie's experience during the Peninsular war, and form a valuable addition to our existing surgical literature.

There is no division into sections or chapters, nor does the arrangement of the lectures appear to have been a precise one. Whatever advantage this may offer the lecturer, and we question if it has any, it certainly is of none to the reader, still less to the reviewer. We will not therefore attempt to present a strict analysis of the work, but select what portions of it, whether facts or doctrine, are either novel or important.

Mr. Guthrie commences by pointing out the uncertainty of the symptoms of injuries of the head, and the equal uncertainty of their diagnosis. After observing, what is well known, that some persons will survive the loss of considerable portions of the brain, while others will sink under much slighter injuries, Mr. Guthrie remarks :—

"The result of my experience on this point is, that brain is more rarely lost from the fore part of the head with impunity, than from the middle part; and that a fracture of the skull, with even the lodgement of a foreign body, and a portion of the bone in the brain, may be sometimes borne without any great inconvenience in the back part. During the war with the United States in 1814, a soldier in Canada was struck by a ball which lodged in the posterior part of the side of the head; the wound healed, and the man returned to his duty. Twelve months afterwards, having got drunk, he fell down in the streets of Montreal, and died. The ball was found on the corpus callosum, where it had made a small hole or sac for itself. After the battle of Toulouse I had three cases, in each of which a piece of the occipital bone was driven in by a ball, which, striking directly upon it, made a hole no larger than the end of the finger: the absence of serious symptoms in these cases insured exemption from operative treatment. One case was, however, peculiar: the part injured was so exactly the size of the ball, and the bone was so clearly to be felt deep in the posterior lobe of the brain, whilst the ball had probably gone beyond it, that I thought it right to recommend the man to have the bone removed. He declined, but begged to have more to eat, which I in turn refused. He had no bad symptoms, and the wound closed in, and had healed when I left him at Bordeaux about to embark for England. It was the recollection of these cases which induced me, after the battle of Waterloo, to recommend, in that of a soldier similarly wounded, that nothing should be done unless symptoms arose demanding the use of the trephine ; as none appeared and the wound healed, the man was sent home to Colchester, where he one day got drunk, and also fell down dead in the market-place. The ball was lodged deeply in the posterior lobe of the brain in a sort of cyst. I have never seen a person live with a foreign body lodged in the anterior lobe of the brain, although I have seen several recover with the loss of a portion of the brain at this part. My experience then leads me to believe, that an injury of apparently equal extent is more dangerous on the forehead than on the side or middle of the head, and much less so on the back part than on the side." 3.

A fracture of the vertex is infinitely less serious than one of the basis. The latter obtains most in civil and hospital practice—the former on the field of battle. Hence, perhaps, one reason for the difference of opinion between army surgeons and those attached to the metropolitan hospitals.

Mr. Guthrie refers to the experiments of Flourens, Mayo, and others, on the destruction of parts of the cerebrum, &c. and to the researches of Dr. Marshall Hall on the excito-motory system of nerves. Applying the facts elicited by these gentlemen to the prognosis of injuries of the head, he concludes that,—" Great severity, and persistence of the symptoms lead to the belief that the part of the brain, or spinal cord on which they depend, is directly injured rather than indirectly affected, and that the result is more likely to be fatal. Permanent insensibility and loss of motion may depend on cerebral mischief only. The loss of the mobility of the iris implies an affection of the tubercula quadrigemina. Convulsions, vomiting, a drawing up of the limb not affected by paralysis, stertor, a difficulty in swallowing, strabismus and relaxed sphincters, show derangement of the spinal functions; which is well marked when tickling the eyelashes causes no closing of the lid, the verge of the anus no contraction of the sphincter, the sole of the foot no motion of the toes."

Mr. Guthrie passes to the consideration of *Concussion.* He comes to the conclusion that the exact condition or lesion of the brain is far from being very clear, whilst he agrees, as all rational thinkers must, with Sir B. Brodie, that " there may be changes and alterations of structure in the brain, which our senses are incapable of detecting."

Speaking of the impossibility of determining what amount of injury is necessary to give rise to fatal concussion, he relates a remarkable circumstance:—" Two men were brought to the Westminster Hospital apparently dead; one had fallen from the dome at the top of Buckingham Palace on the back and head of the other, who was walking unconcernedly below, and who was killed on the spot, although no bones were apparently broken. The man who had fallen from the dome—perhaps the greatest height from which any one has fallen without injury, was quite well on the third day, felt only a little stiff, and left the hospital to return to his work."

We once saw a man brought into St. George's Hospital, who had fallen from the top of one of the houses in Belgrave Square, before the building was completed. The fall had been broken by the scaffolding, and he lit on some loose rubbish on the ground. He was taken to the hospital for dead. Gradually he was prevailed on to open his eyes, move his limbs, sit up, get up, and walk to bed. There was nothing the matter with him.

Mr. Guthrie adds:—

"I once saw a girl of ten years of age fall thirty feet from the parapet of a house on the ground, which was rather soft; I ran to her, thinking she must be killed, but she got up, and ran away roaring and rubbing her bottom, which seemed for several days the only part inconvenienced by the fall. I have read in one of the older authors, however, of a young Dutch girl, who, falling in this way, was so much shook by it and by the rebound, as to suffer afterwards from suppuration on the dura mater at the vertex, requiring the use of the trephine." 10.

Mr. Guthrie goes on to give a full account of the symptoms and progress of concussion. We shall only allude to such points as strike us.

He gives a caution against administering liquids to a patient in this state, before he is well able to swallow. Mr. Andrews mentioned to him the case of a man who was taken to the London Hospital. After drinking he had fallen down stairs, vomited, and died. Nothing could be found of any importance on examination, save some meat in the pharynx, a portion of which had also slipped into the glottis during vomiting, and had suffocated him. Mr. Guthrie has seen a man killed by being made to vomit when lying on his back; and in all cases of insensibility the person should be raised when it is intended he should swallow, and a small quantity only, if anything, should be given at one time.

There can be no question that the act of swallowing is an *excited* act, and being induced by the contact of a substance with the fauces is thenceforward involuntary. But we have only to observe an apoplectic or a paralytic patient to be satisfied of the influence of the brain. Attention and volition are very requisite for the safety and precision of the process.

*Vomiting* is, in general, an early symptom of returning sensibility. But it may wait on extensive lesion. Petit relates the case of a man who died after continued vomiting for seven hours; an enormous quantity of blood was found in the ventricles of the brain. We have all seen vomiting usher in apoplectic paralysis or coma, and it is notoriously a symptom of many organic cerebral lesions. We remember a case of tumor of the cerebellum, in which incessant vomiting was the prominent feature.

We extract a rather long note on the *conditions of the iris.*

"Great stress has frequently been laid by writers on the mobility of the iris, as an indication of concussion, or of compression, or of irritation of the brain. I taught in my Lectures, as early as the year 1818, that the motions of the iris were influenced in three ways; one by the direct stimulus of light, the patient being quite blind; and two by sympathy or indirect influence; the first, with the retina of the same eye when sound; the second with the iris of the other eye, whether the retina was healthy or otherwise. The facts were stated from the observation of these parts in man whilst in health and under disease; and little or nothing has been added to our knowledge on the subject by experimental anatomy. It has, on the contrary, tended to obscure it practically, although it may eventually be useful: for the surgeon would be led into error in the treatment of diseases of the eye, who attended to it alone.

"The optic nerve is probably not a simple but a compound nerve, and possesses the incident and reflex fibres of Dr. Marshall Hall in addition to those for sensation; the former exerting an influence perhaps on the motions of the iris, which is more distinctly supplied with nerves from the lenticular ganglion. When the optic nerve is divided within the cranial cavity, the iris, it is said by Mayo and by Flourens, loses its contractile power, although it may be again excited, and the pupil be made to contract by irritating the root of the optic nerve still attached to the brain. A man may, however, be blind from a defect in the retina or in the optic nerve, and utterly incapable of distinguishing light from darkness; yet the pupils will contract and dilate under the proper influence of light, proving that it is not on the optic nerve, as one of sensation, that these changes depend. The division of the optic nerve within the head commits in all probability a greater and a different injury on the parts than that which takes place from disease. The part of the brain may not be sound in which perception takes place, whilst that part may be healthy to which other impressions are conveyed. Vision may be lost, yet the iris may be movable. The cerebrum may be injured, yet the cerebro-spinal column, and particularly the corpora quadrigemina or upper part may be sound. An injury to the third nerve paralyses the iris. It is said that an injury to either of the corpora quadrigemina does the same. A certain kind of injury to the fifth nerve may deprive a person of sight, but it does not always at the same time affect the motions of the iris.

"None of the changes which take place in the appearance of the iris can then be considered as distinguishing signs of concussion or compression, or of irritation of the brain; they merely imply that a derangement of a particular part has taken place within the head, which may not be perceptible on examination after death, or which may subside and be removed without leaving any permanent defect.

"Dr. Auchinclose has related a case in the Glasgow Medical Journal, copied into the sixth volume of the Medical Gazette, in which, after an injury to the head, he found the left eye was blind, yet the iris acted freely, and the patient recovered.

"Mr. Hancock, when House Surgeon of the Westminster Hospital, examined the head of a woman, a patient of mine, who died three weeks after the receipt of a blow which was considered to have caused only a concussion of the brain. The pupils contracted for several days before her death, separately and conjointly, although the levator muscle of the left eyelid was paralysed, and the eyelids appeared to be nearly closed. An abscess had formed in the base of the skull, implicating and destroying the third nerve of the left side at the point at which it leaves the crus cerebri, which led him to think that the mobility of the iris might continue after the motor oculi or third nerve was separated from the brain. The other muscles of the eye supplied by the third pair were also implicated, and the eye was fixed and the conjunctiva inflamed." 16.

After alluding to stertorous breathing as a symptom of compression, Mr. Guthrie observes that another modification of breathing is equally characteristic and dangerous—it is a peculiar whiff or puff from the corner of the mouth, as if the patient were smoking. This is not an uncommon feature in apoplexy, and is, in fact, a modification of stertor.

Mr. Guthrie also adverts to the slow and laboured pulse which usually waits on pressure or extravasation, but takes occasion to state that many of the largest extravasations he has seen, and many of the most diffused, have been accompanied throughout by a very quick pulse. Yet, wherever he has made pressure on the brain or dura mater during life, a diminution of the frequency of the pulse has been the consequence.

Whilst speaking of the effects of bloodletting in concussion he relates two or three cases illustrative of the bad consequence of pushing it too far.

Perhaps one, communicated to him by Mr. Andrews, of the London Hospital, is as striking as any.

"A young gentleman struck his head against the ground by jumping out of a chaise, which slightly stunned him, but did not prevent his walking home, nearly a mile. He was bled to thirty ounces, but gradually becoming more stupefied, he was bled again to thirty ounces more. This was followed by convulsions, and an increase of the comatose symptoms, for which external stimuli were used with advantage. It was now thought necessary by another surgeon to open the temporal artery, but a small quantity of blood only was abstracted, when the patient died." 21.

Mr. Guthrie offers the particulars of several cases illustrative of the effects of bleeding. His opinions on the point are the following;—

"There is then a time, when the stage of depression is slowly passing into that of excitement, in which it may be doubtful what quantity of blood, if any, should be taken away; but the loss of six, eight, or even of ten ounces can do no harm if they do no good, and their loss may enable the surgeon to form a more accurate judgment of the state or degree of the complaint than he could otherwise have done. When the period of excitement or of inflammation has begun, and the patient, although 'disposed to coma, but when roused is still irrational and impatient,' he is not to be left to await the effects of a blistering plaster or a dose of physic, as has been recommended in such cases, but ought to be bled sitting up in bed to whatever extent may be necessary to relieve the symptoms, or at least to cause a near approach to fainting, for nothing less can relieve such a person effectively, and give him a fair chance for life. The bleeding must be steadily repeated as the symptoms recur until relief is obtained, or until it becomes evident that the powers of the patient cannot resist the inroads of the disease and of the efforts made for its cure. The quantity of blood lost in two or three days is sometimes enormous in powerful healthy men, amounting to 100, 150, and even 200 ounces, with the happiest effect." 22.

There can be no doubt that many a life has been saved by active bleeding. There can also be no doubt that many a life has been destroyed by it. The *juste milieu* is not always easy to be hit, and certainly cannot well be defined. But it is something to put surgeons on their guard and to impress them with the conviction that there are dangers on both sides, and that discrimination is necessary.

Mr. Guthrie relates several other cases, and then proceeds to observe, that "in the less important cases of injury one bleeding will answer the purpose, cupping and leeches may also be resorted to with advantage; but in all very severe ones general blood-letting is the only trustworthy source of relief. It should always be done with effect, the finger examining the opposite pulse and regulating the amount to be taken away. At an early period of concussion the quantity should not be large: it should increase with the urgency for its abstraction, and diminish with the frequency of the repetition, being always, however, carefully regulated by the effect. The inability of blood-letting to overcome the disease will be shown by the increase in frequency of the pulse, its diminution in power under slight compression, its greater softness, together with the persistence of the other symptoms.

"It is in these cases that repeated small bleedings, to the amount of six or eight ounces, ought to be resorted to, and when it is doubtful whether the loss of blood can or cannot be borne; they may then be considered not as curative but as explorative measures, although they may sometimes prove very effective."

In these cases, calomel given early and rapidly, particularly if combined with opium, may be "decisive." We apprehend that, in head cases, the general employment of opium, even in combination with mercury, is not free from objection. Not that we would proscribe opium, but we think that calomel alone, or in connexion with James's powder, is, in many instances, preferable. Blisters, at a later period, shaving of the hair, cold lotions or ice to the scalp, are all, of course, recommended. But Mr. Guthrie does not mention, what we have seen of great utility, the application of a blister to the scalp and dressing the surface with mercurial ointment. This measure is necessarily applicable to the later stage of inflammation of the brain or of its membranes, after depletion and the internal exhibition of mercury.

Mr. Guthrie refers to the insensibility produced by inebriation, and the possibility of its being mistaken for the effects of injury. But the odour of spirits is demonstrative of the fact, and the stomach-pump is the remedy. "There was a man in the neighbourhood of the Westminster Hospital formerly, who frequently got drunk and as generally fell down apparently insensible, and was brought to the hospital. The first time there was some doubt about the case, but never afterwards; and he became so fearful of the pump as to take care that he got drunk only when at a distance from his home." The difficulty however, in some cases, is this, to determine when an intoxicated person has fallen, whether cerebral injury may not be combined with the intoxication.

"There is another kind of case of infinitely more importance; it is when mania supervenes on the injury, from the consequences of which it has often been undistinguished. It occurs only, I suspect, when the sufferer has an hereditary predisposition for insanity, and rarely unless he has shown some previous symptoms of such derangement. The first case I saw of the kind was in a soldier after the battle of Salamanca, who had suffered a slight injury of the head, and my suspicions as to the nature of the case induced me to examine the brain after death, when nothing could be found to account for it. The second occurred many years ago in the Old Westminster hospital: the man had fallen from a moderate height, and suffered from the ordinary symptoms of concussion through the first and second stages, when they assumed those attendant on mental derangement. He sat up, talked irrationally as well as incoherently, required some restraint to keep him in bed, owned to no complaint, would eat as well as drink anything that was offered to him; the pulse never ranged above 88, and all the ordinary functions were regular. He died at the end of three weeks apparently exhausted, and nothing peculiar could be perceived in the brain. This man might possibly have recovered under the use of opium, which I have since found of great utility in several cases; the preparations I prefer are those of morphia, which seem to cause less headache and less confinement of the bowels, although they sometimes give rise to nausea and sickness, when the dose is too large." 29.

Our readers are aware that morphia has been much lauded in cases of mania, and does certainly in some instances prove serviceable. But we cannot help thinking that more harm than good has been done by it, and that there is an extravagance in this direction, as well as on that of depletion.

Mr. Guthrie touches on the more remote effects of concussion upon the brain and its membranes. The patient may suffer little, or not at all, at the period of the accident, but subsequently suffer from pain in the head or other symptoms. He relates a case in which very large bleedings were practised for such symptoms and procured perfect relief.

The convalescence after injuries of the head requires extreme care. Relapses are frequent, and undermine the health. " In many instances," says Mr. G. " and particularly among poor people subject to privations and of irregular habits, in whom an injury of the head has not originally been of any apparent importance, such a state of irritation, combined with debility, is very difficult to manage, and requires a combination of local as well as of general means for its cure. A few leeches and blisters may be applied alternately over the part affected, with great advantage, and a mild nourishing diet with gentle alteratives and tonics will expedite the cure, especially when aided by perfect repose and a fresher atmosphere. In persons of a higher station, who rather suffer from casual irregularities, I have found an issue in the arm, which establishes a gentle but permanent drain, a most efficacious remedy; and I am in the habit of recommending its adoption in all cases of affection of the head among elderly persons, in which any material or long-continued inconvenience has been suffered."

Mr. Guthrie passes to the subject of extravasation of blood within the cranium and compression, and seems to lean to the opinion that the brain *is* compressible. But the knotty question is not much smoothed down, and we must admit that our author leaves it pretty much as he found it.

Our author gives a copious account of the symptoms of compression. The only point to which we shall allude is the relation between the side of the brain affected and the side of the body paralysed. After quoting opinions, Mr. Guthrie observes, that " Burdach found in 268 cases of lesion of one side of the brain, that ten presented paralysis on both sides of the body, two hundred and fifty of one side, and of these, in fifteen the paralysis was on the same side as the injury. The convulsions were in twenty-five cases on the same side as the disease; in three cases on the opposite side. In cases of lesion of one corpus striatum, there were in thirty-six instances paralysis of the opposite side, and six with convulsions of the same side, and in no instance convulsions of the opposite side. In twenty-eight cases of cerebral lesion of one side the muscles of the opposite side of the face were paralysed, in ten cases those of the same side. Paralysis of the eyelid was in six cases on the same side, in five on the opposite side. Paralysis of the muscles of the eyeball occurred in eight cases on the same side, in four on the opposite. Paralysis of the iris in five cases on the same side, and five on the opposite; the tongue being generally drawn towards the paralysed side of the face." Mr. G. alludes to several other opinions, but to mention them is sufficient to expose their hollowness.

We extract Mr. Guthrie's observations on convulsive movements of the limbs, after injury of the head—a subject full of difficulty. After remarking that they have been known from the earliest antiquity to occur, and it has been also known that they generally affect the side opposite the paralysed one.

" When the paralysis is not complete, I have frequently seen that side affected by slight convulsive twitches, whilst the other suffered from well-marked spasms; leading to the belief, that whilst paralysis is an affection of only one half of the brain of the opposite side, or of half of the spinal marrow of the same side, convulsions are the effect of a more general irritation, capable however of being confined to a part; for partial convulsive motions do very frequently occur without any paralysis accompanying them on the opposite side, and I have not seen these convulsive actions occur, as far I can recollect, where both sides have been paralytic from injury of the head, although spasms and twitches are symptoms of daily occurrence in paraplegia from disease of the spine. I have met with several cases in which the convulsions have ceased and the patients recovered after the removal of a portion of bone which was irritating the brain; but convulsions have generally been the forerunners of death when the seat of injury was unknown and this assistance could not be given. When they occur in cases apparently of pure concussion, accompanied by inflammation of the brain or its membranes, and the patient recovers after many days of the strictest antiphlogistic treatment, it is possible that the brain may have been lacerated and the cure have been effected by adhesion. Convulsions, it must be remarked, are among the most common symptoms of inflammation of the membranes of the brain, without any such lesion of its substance, although they are frequently wanting. They may be expected to take place about and after the fifth day in injuries of the head, when inflammation of the brain or its membranes is about to extend to or become continuous with the neighbouring parts, and may be more or less severe, varying from a state of partial trembling of a limb to that of general agitation and restlessness of the body generally; from a slight irregular movement of the eyelids, or muscles of the face, to the more marked spasmodic startings of the whole of one side, grinding of the teeth, and contraction of the limbs. Sir B. Brodie has well shown in his memoir, that they may exist at a late period independently of inflammation, 'being aggravated by any additional abstraction of blood, and subsiding on the patient being allowed to take some more substantial nourishment than that which had been allowed him previously.' They would seem in these cases to be dependent on the same or similar causes to those which gave rise to them after the loss of too great a quantity of blood in the first instance, and to be relieved or removed in a similar manner. It is far different with those convulsive movements which, at a late period, became nearly permanent or rigid spasms, resembling tetanus, in which the body is drawn in different directions forwards, backwards, or to one side. They are for the most part the forerunners of death; fortunately they are seldom present except in very hot weather,* and are not even then of frequent occurrence. Examination after death in such cases has shown nothing discoverable beyond inflammation of the pia mater, and an effusion of fluid, generally purulent, on the surface of the brain or in its ventricles, or between the pia mater and tunica arachnoides." 50.

Amongst other cases bearing on the point, Mr. Guthrie relates an interesting one which Mr. Keate took him to see in St. George's Hospital. It seemed to be an instance of injury of the head and paralysis on the same side. The paralysis although positive, was not so complete as to render the patient quite incapable of moving the arm and leg, which were frequently convulsed, although the convulsions which were observable in both were more marked on the opposite or left side. But on dissection the apparent anomaly was cleared up. For, the most serious injury was a fracture of the right parietal and temporal bones, ex-

* "The most remarkable cases I have seen, occurred after the battle of Salamanca, when the weather was very hot and the hospitals for the most part were crowded."

tending to the petrous portion of the latter, and beyond it, which, with the rather large extravasation of blood under and in the course of the fracture, appeared to be sufficient not only to destroy life, but to have caused paralysis of the left side, which it did not do. Another extravasation, rather less in quantity, had however taken place under the upper and anterior portion of the left parietal bone, which enabled him fully to account for the paralysis which took place on the right side, and which nothing but a *post-mortem* examination could have made known.

Mr. Guthrie, like all surgeons of experience in the present day, is no advocate of the trephine. In cases of fissure of the cranium, or of simple fracture, if no symptoms of compression exist, it is improper to resort to it. If symptoms come on, it will be time enough to act upon them.

Mr. Guthrie observes:—

"After the receipt of a severe blow or a gun-shot fracture of the head, which has not even stunned the person at the moment, he may walk to the surgeon, and be dressed, and converse with his fellows as if nothing had happened; yet in a short time he becomes heavy, stupid, drowsy, unwilling to move, with a slow pulse and a pallid countenance. Inflammation has not yet had time to set in, and extravasation has not always taken place. If the loss of a moderate quantity of blood should relieve such a person, it shows that congestion had occurred, perhaps on the surface of the brain under the injured spot; recovering from which by the unassisted efforts of nature, he would still be liable to inflammation. I have repeatedly seen a sharp bleeding from an incision made to allow a complete examination of the part in such a case, cause the restoration of the patient to his natural state. A return of untoward symptoms during the progress of the case does not always indicate essential mischief, and will be removed, if of a temporary nature, by a further moderate bleeding, by purgatives, and by greater restriction in diet, through irregularities in which, these secondary attacks most usually occur. If the loss of blood should not relieve the symptoms, the case is probably complicated by an extravasation having taken place between the dura mater and the bone, or even in, or on the surface of the brain." 58.

Mr. Guthrie adverts to injury of the parietal bone occasioning rupture of the middle meningeal artery, and effusion of its contents. If the case is recognised, it is, of course, proper to trephine. But Mr. Guthrie observes:—"Experience has demonstrated, that persons have recovered after large coagula have been removed; but in all these cases the brain had not lost its resiliency, and had been seen to regain its natural level on the removal of the depressing cause. I have several times seen the depressed brain gradually recover its natural position, and the person open his eyes, and recognise and speak to those about him; but I never saw the symptoms mitigated, or the persons in any way relieved, when the brain remained depressed after the blood had been removed." He relates some cases illustrative of this position.

Serious, and commonly fatal, as fracture of the base of the cranium is, it is not universally so. From several cases, intended to illustrate this, we select one, communicated to our author by Mr. Keate.

*Case.*—"A young gentleman, eleven years old, fell down a flight of kitchen-stairs, on a stone pavement on his face, in September 1839; his nose bled considerably, and appeared to be flattened and a little out of shape: he complained only of the pain of his nose, which in a few days quite left him. Three weeks afterwards an abscess formed behind the left ear of the size of a small hen's egg, which was opened and healed. He then went into Devonshire, and remained some months apparently in perfect health, when, without any cause which his friends could assign, he every night suffered from retching without actually vomiting, which gradually subsided, and he afterwards passed a good night. In December 1840 he died after a short illness, his death being preceded by all the symptoms of hydrocephalus, and Mr. Norton furnished Mr. Keate with the following report of the *post-mortem* examination. The width of the head from ear to ear was greater than usual in a child of his age; the pericranium was easily separated from the left parietal bone, which appeared discoloured; the dura mater appeared more vascular than usual; the sinuses were full of blood; there was considerable effusion between the dura mater and arachnoid membrane, and some coagulated lymph around the tract of the optic nerves, which were soft, and readily torn across; a quantity of serous fluid escaped from the ventricles, of which six ounces were preserved. On removing the brain a small abscess was discovered upon the sella turcica, and the bone in front was very rough. A fracture or fissure was also perceived running across from the temporal and between the sphenoid and ethmoid bones, and which no doubt was occasioned by the fall he had received fifteen months before." 70.

Our author alludes to a very serious symptom, after injuries of the head —the discharge of a watery fluid from the ear. This, probably, comes from the sac of the arachnoid membrane, and is indicative of great danger. In these cases, the principal fracture is usually in the direction of the petrous portion of the temporal bone, and towards the body of the sphenoid. Although the extravasation of blood, may take place from a rupture of the lateral sinus, it is as frequently found under the middle, or one of the other lobes of the brain, accompanied by laceration of its substance. Mr. G. has seen the fracture pass across the carotid canal, and the extravasation caused by the rupture of the artery.

Fracture and depression of the inner table of the skull, without fracture of the outer, next occupies our author, and he quotes many authors and their cases. But perhaps none are so satisfactory as De la Motte, who supposed that, when the inner table was broken without the outer one, the patient might be aware of the fact, by the peculiarity of sound which followed the blow, resembling that given out by a broken pot when violently struck, and he relates a case in illustration of this idea. It must be owned that the diagnostic sign is likely to prove very valuable.

Mr. Guthrie, however, very justly, as it seems to us, discountenances the notion that depression of the inner table, without fracture of the external one, is a common accident, or one warranting interference. He remarks :—

"I therefore think it safe and reasonable to come to the conclusion, that although these things have happened, they will rarely occur again. I have never, in the great number of broken heads I have had under my care on many different, and grand occasions, actually known the inner table to be separated from the outer, without positive marks of an injury having been inflicted on the bone or pericranium, however slight that injury may have been; and although it is not possible to doubt the fact of fracture of the inner table having occurred, it is very desirable in a practical point of view not to bear it in mind; for if a surgeon should be prepossessed with the idea that the inner table might be so readily fractured, and separated from the diploe placed between it, and the outer table, and thus cause irritation or pressure on the brain, few persons who had received a knock on the head, followed by any serious symptoms, without fracture or depression, would escape the trephine, and the worst practice would be again established. An operation should never then be performed under the expectation that such an accident may have happened, unless it is apparently required by the urgency of the symptoms indicating compression or irritation of the brain, which cannot be relieved by other means." 79.

Mr. Guthrie admits, indeed, that a blow on the head will frequently detach the dura mater from the inner table by rupturing its vessels, and thus give rise to compression or irritation of the brain from the effusion of blood or the formation of matter, and that the inner table may become diseased from the same cause, and be the cause of ulterior mischief. If so, there are ulterior means of treatment. We shall quote one or two of the several cases cited by Mr. Guthrie.

*Case.*—" Mr. C. Trye of Gloucester, relates a case of an injury of the internal table of the skull successfully treated in the year 1786. Nine weeks after the accident, the external table of the right parietal bone being evidently dead, the trephine was applied, and he found then that the greater part of the internal table had been removed by absorption, and that granulations were springing up from the parts beneath, but whether they were from the dura or pia mater or brain could not be accurately ascertained. The man recovered." 78.

*Case.*—" Dease trephined a young man nine months after he had received a blow on the upper part of the os frontis, which caused him great pain in the head, rendering him in general incoherent in his speech, and infirm in his limbs. The wound was not quite cicatrized. On examination Dease found a depressed fracture larger than the breadth of a sixpence, which he removed with a large crown of a trephine. The three subsequent days he extracted ten pieces of the inner table, which had been driven into the brain. The man left the hospital in about three months in perfect health." 81.

But perhaps the following is the most extraordinary case of all. Whether or not it is a good sample of Continental surgery we leave it to others to say.

*Case.*—" A man 36 years old received a blow from a stone on the left parietal bone, from which he thought he had recovered on the sixth day: it was however followed by such frequent, and violent attacks of pain as to render him unable to work; and after all other means had been tried in vain, he was trephined. Nothing abnormal being found, Walther thought he would replace the circular piece of bone he had removed, which he did, and the replacement was not followed by any severe symptoms. At the end of three months, during which time the pain in the head went away, he saw a loose piece of bone at the bottom of the wound, which had not healed, and on removing this, he found it was a part of the external table of the replaced bone. The wound soon healed after this, and the patient recovered (in defiance of the doctor)." 83.

Passing over many other cases, we are induced to insert one which occurred to Mr. Guthrie himself.

*Case.*—" M. A. Farnham, aged twenty-three, a stout healthy-looking girl, received a blow, two years ago, from a stone falling from a door-way under which she was passing, which struck her upon the left side of the head at a spot an inch anterior to the parietal prominence, the weight of the stone, and the space through which it fell, making the estimated force with which it struck the head equal to sixteen pounds. The immediate effect of the blow was insensibility, followed by acute fixed pain in the head, which has ever since continued to mark the seat of injury. A week after the receipt of the blow she began to lose the power of moving of the right arm, there being however no loss of sensation or any disturbance of the cerebral functions.

"During the following twelve months her symptoms remained unchanged, and this period was spent in Guy's, St. Thomas's, Westminster, and St. George's Hospitals; but having derived no relief whilst in any of these institutions, she became an out-patient under the care of Dr. Roe.

"After the lapse of a few weeks the paralysis of the arm suddenly increased, sensation still being unaffected, and she experienced no further change in her condition until after eleven months, when she was again admitted into the hospital, her symptoms then being the following:—the arm and leg of the right side quite paralytic, the former, which had previously been flaccid, having now become remarkably rigid, and its temperature being below that of the opposite side; vision, particularly of the left eye, imperfect, the pupils however acting naturally; hearing on that side also affected; memory bad; respiration frequently slow and almost stertorous; the countenance assumed a dull heavy expression, and she manifested an unusual tendency to sleep. All the ordinary remedies having failed to relieve these symptoms, Mr. Guthrie was requested to see her, and the operation of trephining was eventually agreed upon.

"*April* 1*st*, 1841.—Mr. Guthrie this day removed a disc of bone from the exact point in the parietal region to which she referred the pain. The portion of bone presented no evidence of disease; its thickness varied from two and a half to four lines, the latter measurement corresponding to the part most distant from the sagittal suture: the vessels of the diploe bled freely, the dura mater was quite healthy, and without any very evident motion.

"On visiting her *an hour* after the operation, she raised the previously paralytic arm several inches from the bed, and was able to bend, and extend the fingers. The pain in the head was considerably less, and her countenance, before dull and heavy, was now remarkably animated. Sensation had returned in the arm, and partially in the leg. Her pulse was calm, and skin cool.

"Ten hours after the operation she was attacked with rigors, followed by pyrexia and all the symptoms of commencing inflammation of the brain. By the immediate abstraction of blood, which was three times repeated during the succeeding twelve hours whenever the pain in the head or the force of the circulation increased, every bad symptom was removed. In the course of three days the paralysis had completely disappeared, sight and hearing again became perfect, and after passing through a speedy convalescence, she quitted the hospital completely recovered." 85.

Certainly, the preceding is a singular case. We are told that the girl has since had some relapses of pain and uneasiness in the head, and that she is of a very hysterical temperament. Could the symptoms have been of this character? We know how many forms they assume, and how they sometimes, by a sort of caprice, yield to operations or to remedies which are generally unadvisable. The absence, in this instance, of any perceptible lesion, the sudden recovery without any assignable reason, and the temperament of the girl, render this not altogether improbable. At the same time there is a per contrà, which throws a great degree of obscurity upon the case.

Mr. Guthrie directs attention to a peculiar fracture of the inner table. It occurs from the blow of a sword, hatchet, or other clean cutting instrument, which goes through scalp and skull into the brain. It is usually supposed that in this instance there is no fracture, but an incised wound of the bone. When the outer table only is divided, it should be so treated; and so it should be when the diploe is involved. But "when the sword or axe has penetrated as far as, or through the inner table, the case is of a much more serious nature; for this part will be broken almost always to a greater extent than the outer table; and will be separated from it, and driven into the membranes, if not into the substance of the brain itself; the surface of the bone showing merely a separation of the edges of the cut made into it. These cases should all be examined carefully. The length of the wound on the top, or side, or any part of the head which is curved and not flat, will readily show to what depth the sword or axe has penetrated. A blunt or flat-ended probe should in such cases be carefully passed into the wound, and being gently pressed against one of the cut edges of the bone, its thickness may be measured, and the presence or absence of the inner table may thus be ascertained. If it should be separated from the diploe, the continued but careful insertion of the probe will detect it deeper in the wound; a further careful investigation will show the extent in length of this separation, although not in width; and will in all probability satisfy the surgeon that those portions of bone which have thus been broken and driven in, are sticking in or irritating the brain. In many such cases there has not been more than a momentary stunning felt by the patient; he says he is free from symptoms, that he is not much hurt, and is satisfied he shall be well in a few days."

Mr. Guthrie relates some cases in point. Perhaps the first is as much so as any.

*Case.*—"An officer was struck on the head in Halifax, Nova Scotia, by a drunken workman with a tomahawk, or small Indian hatchet, which made a perpendicular cut into his left parietal bone, and knocked him down. As he soon recovered from the blow and suffered nothing but the ordinary symptoms of a common wound of the head with fracture, it was considered to be a favourable case, and was treated simply, although with sufficient precaution. He sat up, and shaved himself until the fourteenth day, when he observed that the corner of his mouth on the opposite side to that on which he had been wounded was fixed, and the other drawn aside; and that he had not the free use of the right arm so as to enable him to shave. He was bled largely, but the symptoms increased until he lost the use of the right side, became comatose and died. On examination, the inner table was found broken, separated from the diploe, and driven through the membranes into the brain, which was at that part soft, yellow, and in a state of suppuration." 87.

The application of the trephine is, of course, the proper practice. The following is an instance of it.

*Case.*—A soldier was wounded by a sword on the top of the head. The bone was apparently only cut through, but the inner table was depressed, and felt ragged under the probe. On the fourth day, the symptoms of inflammation increasing, and not being relieved by bleeding, Mr. G. removed a central portion of the cut bone by one large crown of the trephine, and took away several small pieces which were sticking into the dura mater, after which all the symptoms gradually subsided.

Mr. Guthrie quotes a case and an opinion of Sir Philip Crampton's.

The case we may omit—the opinion is to the effect that where, in fractures of the kind we have been discussing, a fragment of inner table is driven into the brain, it is better not to trephine at first, for, argues Sir Philip,

"The operation, in the first instance, would have been an additional violence to parts already severely irritated, and consequently an additional source of inflammation. It would besides have removed all support from the wounded brain, a great part of which would (it is probable) have escaped through the opened dura mater. If the patient escaped these first dangers, then came the danger of hernia, or rather fungus cerebri—one of the most frequent and dangerous consequences of wounds of the dura mater." 90.

But hear Mr. Guthrie on the other side:—

"It appears to me that too much stress is laid upon a difference which is supposed to exist in the danger of trephining a man on the first or on the seventh day after an accident, and that an error may be committed in believing that the trephine is a more dangerous instrument on the first day than on the seventh. The question here is not whether the man is to be trephined or not? but which will be the best and safest day or time to do the operation? I do not hesitate to say the first day. I believe the violence to be greater when done on parts already in a state of inflammation than when they are sound. I am quite satisfied, that when the inner table is sticking through the membranes and into the brain itself, the individual will in most cases ultimately die miserably of the accident if not relieved by art; and that it is less safe to let him designedly run the certain risk of cerebral irritation, which when once excited is often indomitable, than to remove the cause, and so endeavour to prevent the evil. If the cerebral irritation only manifested its effects on the surface of the dura mater by causing suppuration there, I might yield my opinion, but as I know that it often gives rise under these circumstances to the formation of matter on the surface of the brain, and under its membranes, where it is generally deadly, I cannot assent to that which may be called 'la chirurgie expectante.' Lastly, I do not think that there is more danger of a hernia cerebri when the operation is done early than when it is done at a later period; on the contrary, I think the patient has a much better chance of escape from hernia cerebri, and from all other evil, when the local and the general treatment are alike immediately decided, and efficient." 92.

There is much to be said then on both sides. Nay, Sir P. Crampton's opinion is reinforced by Mr. Colles, who, in reference to another case, remarks—"In very small depressed fractures (such as may deserve the name of punctures of the bone), where a depressed bit of bone is sunk into the brain, it will perhaps be prudent to postpone the operation for a few days. For if the operation be performed immediately after the receipt of the injury, and if we attempt to seize the depressed fragment, the first touch of the forceps sinks it more deeply into the brain; portions of the brain, from the softness of its texture, rise up and conceal the bone both from our sight and touch, whereas, if we defer the operation for a few days, we give time for the adhesive inflammation to take place; this circumscribes the depressed piece, hardens this spot of the brain, and thus enables us more easily and certainly to lay hold of the fragment of bone."

For our own parts we feel inclined to agree with Mr. Guthrie. If symptoms are likely to occur, and if the trephine will probably be necessary, it seems more consistent with reason and analogy to use it on uninflamed than on inflamed parts, as a preventive measure than as a curative one. If, indeed, it could be argued that, in a large proportion of cases, symptoms are not likely to ensue, the case would then be different. Mr. Guthrie reasons both ingeniously and forcibly upon the matter:

"It is necesssary," he says, "to recollect that the brain appears to be insensible, or nearly so, when first exposed; and it has rarely occurred to me to see a serious convulsion, or anything beyond vomiting take place on the removal of a piece of bone from the brain; nor do I suspect any difficulty will be found in removing such small fragments as can be seen, with a pair of forceps duly adapted for the purpose. It is impossible to say at what period of time the brain becomes irritable, and no longer admits of being touched without convulsive movements ensuing; but whenever this state of irritation has commenced, and its existence is proclaimed by the excitement which takes place on touching the fragment of bone, the surgeon should at once desist from all attempts to remove the foreign body. The brain under ordinary circumstances is, I conceive, much more likely to recover from an injury, all foreign or irritating matters being removed, than when also suffering from their presence. Should I be mistaken on this point, the opinion generally entertained of the propriety of removing extraneous bodies from wounds in general, must I imagine be erroneous. It is very inconvenient to remove a granule of iron which has been implanted in the cornea when the eye is irritable, and particularly when the surgeon has not a sharp-pointed instrument to lift it out with; it will doubtless be more easily removed when suppuration has taken place, but the cornea will be in a much worse state. There is in fact no comparison between the two modes of proceeding; and I suspect it will be found to be much the same with the brain as with the cornea.

"The establishment of the principles which ought to regulate the practice of surgery in cases of fracture and depression of the inner table of the skull is of the greatest importance, and it is on this account that I have quoted so many authorities on the subject. The principle being laid down that it is right and proper to examine all such wounds with a blunt flat probe, in order to ascertain if possible whether the inner table is depressed and broken; the question necessarily arises, what is to be done when such depression and breaking down of the inner table is ascertained to have taken place? There can be no hesitation in answering; that in all such cases the trephine should be applied, although no symptoms should exist, with the view of anticipating them. The old doctrine, it may be said, in regard to fractures generally, is revived in these cases, but on a principle with which our predecessors were not sufficiently acquainted. A patient very often survives a mere depression of the skull; he may, and occasionally does survive, a greater depression of the inner than of the outer table; but I do not believe that he ever does survive and remain in tolerable health, after a depression with fracture of the inner table, when portions of it have been driven into the dura mater. If cases could be advanced of complete recovery after such injuries, I should not consider them as superseding the practice recommended, unless they were so numerous as to establish the fact, that wounds of the dura mater and brain, by pieces of bone, are not extremely dangerous. I have referred purposely to many cases in which a cure was effected after a lapse of time, by the bone being removed; but they rather support than invalidate the principle I have inculcated. There are great objections I admit to the trephine being applied in ordinary cases of fracture, which are not attended by symptoms of further mischief; but the nature of the cases which I have particularly referred to, having been ascertained, I maintain that the practice should be prompt and decisive in every instance in which the surgeon is satisfied that there is not merely a slight depression or separation of the inner table, but that several points of it are driven into the dura mater. If one trephine will suffice, the central point being applied close to the edge of the middle of the wound in the bone, it should be applied there; but if the cut be longer, and the spicula of bone extend upwards and downwards in its length, a small trephine should be applied as near each end as may be judged advisable, and one edge of the cut bone should be removed by the straight saw, of which Paré and Scultetus made such use in ancient times, and which Mr. Hey of Leeds revived in modern surgery; or the small straight saw may be used alone, if the object of removing a portion can be attained without the trephine. By these means sufficient room will be obtained to remove the broken portions of bone which are irritating the dura mater, and brain." 95.

When the fracture of the cranium is from a sharp or weighty instrument impelled in a horizontal direction, the derangement of the inner table just noticed may not occur. The nature of the injury in such cases can be more readily perceived, and the broken portions of bone removed. Hey's saw may be necessary, and the wound should be simply treated.

"When," says Mr. G. "a portion of bone is as it were sliced off with the scalp, and adheres to it firmly, the scalp and bone should be re-applied; and the cure will often be effected without difficulty. When the portion of bone cut off, and hanging to the scalp which is turned down, has but little adherence, it had better be removed." 95.

Several cases are related. But the following particulars are, perhaps, the most interesting.

"In the museum of the Royal College of Surgeons there are ten skulls which have suffered from very severe slicing cuts. They appear to have been collected from the burial-place of some establishment for invalid soldiers in Germany. The portions of bone thus sliced, and they are large pieces, were once detached, and afterwards reunited a little out of their proper places, so that the points of union and of separation can be distinctly seen. These fissures are all in a certain state of progress towards being filled up by bone, and the patients must have lived some months, if not years, after the receipt of their respective injuries; for bone is deposited apparently with difficulty, and most carefully in all such cases, so as not to irritate the membranes of the brain. The opening in the first instance is filled up by granulations, over which a thin skin is formed, this afterwards became firmer and harder, being in some cases, where the trephine has been used, a thin but strong membranous expansion extending from one edge of bone to the other. In others it is thicker and more solid, and in a few instances osseous matter is deposited in its circumference so as in part to fill up the opening; the edges of the bony circle made by the trephine becoming gradually thinner as they appear to grow inwards. It is rare that an exfoliation does not take place from the edges of the cut bone, or from the circle made by the trephine. It has been occasionally observed after death, that the circular cut edge of the bone does not become thin in the manner described, but that a sort of ridge forms around and within it, which was thought to be the cause of death in some persons who died suddenly, and in whom no other derangement of structure could be perceived." 97.

Mr. Guthrie is an advocate, of course, of union by the first intention in scalp-wounds, unless the integument has been excessively bruised. Speaking of erysipelas of the scalp, he directs attention to diffuse inflammation beneath the occipito-frontalis, and to the necessity for prompt and free incisions. All surgeons are agreed upon this head. We are not aware that the following practice is so universal.

"Erysipelatous inflammation is more apt to follow punctured wounds in persons who live, or have lived irregularly; and the moment the parts around the cut or puncture have become puffy, the surface of the wound changing from a red to a yellowish colour, with a thin discharge instead of good pus, an incision should be made through them, and repeated in different places as often as may be found necessary. It relieves the tension, and prevents the quickened pulse, the irritative fever, the delirium which would follow, and which neither bleeding, purging, nor the other constitutional remedies which the state of fever may indicate, will remove. If it should be neglected, suppuration and sloughing will extend under the tendon of the occipito-frontalis, or the fascia of the temporal muscle, as the case may be, and the greatest danger will be incurred. Mr. Pott, and many of the older surgeons, were, it is but just to say, aware of the value of incisions in such cases; and Dessault derived the greatest advantage from emetics and purgatives, the use of which is deserving of the greatest attention." 102.

Mr. Guthrie dwells on the great distinction that obtains between depression of the cranium in children and in adults. In the former, the inner table does not break so readily, the brain bears pressure better, and the level of the bone is gradually restored. He believes that, for the last twenty years, the greater number of successful cases of recovery from depression or fracture of the skull, that were not trephined, were in young persons. The following case is a marked one.

*Case.*—"Twenty years ago a small child fell over the banisters of the second floor in a public-house at the top of the Haymarket. I saw it as soon as possible afterwards, lying on the bed, motionless, senseless, breathless, with a hollow in the parietal bone that would have held half of a small orange, and I thought it was dead. In a short time it gave a gasp, another followed at an interval so long as to excite surprise, and a third shortly afterwards led to some hope. The motions of the heart and the pulse, which were only now to be felt, being equally irregular and defective. It gradually recovered, and the next day breathed regularly, could speak, and answer shortly, although apparently otherwise stupid and restless. Pulse 90, and regular. Cold lotions were applied to the head. The loss of a little blood by leeches with some smart purgatives gradually removed the unfavourable symptoms, and the child began to walk about, with a hollow in the side of the head which exceeded anything I had seen before, and it was several weeks before the skull regained its level. The same thing then takes place in the bending of the flat bones of the skull in young children, which is so often observed in the long ones at the same period of life."* 103.

Our author now approaches a knotty question—the essential difference between a simple and compound fracture of the cranium. Our readers are aware that Sir Astley Cooper insisted much on this distinction, and advised that, in compound fracture of the cranium with depression, the bone should be raised by the trephine, or otherwise, whether there were symptoms of pressure or not. Mr. Guthrie's ideas upon this subject differ not only from those of Sir Astley Cooper, but from those of a large number of surgeons of the present day.

---

* "Avellan says that a girl of fourteen had a depression of the right parietal bone from a blow, which gave rise to mental derangement, amounting almost to imbecility, for three months; at the end of which time the depressed bone gradually resumed its level, and the girl completely recovered. In Quesnay, Mémoires de l'Académie de Chirurgie de Paris, tome i."

"The difference between a simple and a compound fracture of the leg is often considerable, it is more often dependent on degree: and when the fracture is nearly transverse, and the skin cleanly divided, the difference between it, and a simple fracture of the same part, is little more than one of time. I suspect this to be the case with an injury of the head, and my experience induces me to believe that the difference between the two states in fractures of the skull has been much exaggerated; so much so, that I place no reliance on the supposition that there is more real danger in a case of fracture with depression in which the scalp has been divided, than when it has been only bruised, and not divided; and I apprehend that in all cases in which a fracture with marked depression is known to have occurred in an ADULT, it is the best practice to divide the scalp, and ascertain the nature and extent of the depression.

"If the result of a great number of comparative trials should prove in favour of never, under any circumstances, raising a depressed portion of bone in an adult, but of leaving it to the efforts of nature, an incision in order to ascertain the state of parts below ought not to be made; but as such result is not likely to be obtained, according to my observation and experience, the practice recommended appears to be the best." 104.

We apprehend, that most surgeons are now in favour of *not* cutting down on depressed bone, unaccompanied with wound, *if there be no symptoms.* If there are symptoms, all surgeons, we suppose, *would* cut down. Mr. Guthrie's rule, *always* to cut down in an adult does appear to us too absolute, and calculated to lead to abuse of the trephine. Such, at least, is our impression. Whether the rule of Sir Astley Cooper is not too absolute also, we will not take upon us to determine. We are rather inclined to think that it is; and in a slight case of compound fracture of the cranium, with depression but without symptoms, we fancy we should pause before we used the knife.

But to return to Mr. Guthrie, and his opinions with regard to the trephine—

"The cranium," he proceeds, "together with the fracture and depression, being exposed, the question whether the trephine should be applied or not, is now to be determined. If the operation by the trephine, or that of sawing a piece of bone out of the head, was not in itself dangerous, there could be no hesitation about its use; but it is a dangerous operation, especially in crowded hospitals, and ought not to be resorted to when it can be avoided. I am of opinion, that if any ten healthy persons were trephined in an hospital, one would in all probability die from the effects of the operation; and that three or four more might have a narrow escape from the inflammation of the brain and its membranes, or the other consequences which would probably ensue. It is not the admission of air, which has been even lately supposed to do mischief, that is to be dreaded in these cases, but the same kind of irritation which often follows the abstraction of a piece of bone under other and more ordinary circumstances at a later period of time." 105.

From several illustrative cases, we select what seem to us most characteristic. The following shows the time that a ball may lodge within the cranium with trifling symptoms, and their ultimately severe or fatal character.

*Case.*—"Thomas O'Brien, 28th regiment, aged twenty-three, was wounded by a musket-ball on the 16th of June at Quatre-bras; the bullet penetrated the occipital bone below and to the right of the junction of the lambdoidal and sagittal sutures. On his arrival at Colchester, the wound was healthy in appearance and healing rapidly. It appeared from his own account that for some hours after the injury he was totally deprived of sight; since that time he has been constantly more or less affected with headaches, for which he has been prescribed occasional cathartics and low diet. He has been also affected with pain and weakness in both eyes, but more particularly in the right. While at Brussels and during his progress to Ostend he lived very irregularly, and was frequently intoxicated; the external wound was entirely healed on the 20th of July, and no suspicion existed that the ball was lodged in the brain. On the 25th matter was perceived under the scalp, and was yesterday evacuated. To-day, the 27th, he complains of increase of headache; pulse small and quick. V. S. ad ℥vj. Haust. cathart. statim. 28th. In the course of this day his symptoms have become very urgent; he is restless with a very quick pulse; an extensive crucial incision was made in the site of the original wound, and now for the first time it was discovered that the ball had penetrated the brain; several loose pieces of bone were extracted; a considerable quantity of arterial blood was suffered to flow from the small vessels divided in the incision. His bowels had been well opened by the cathartic. The most vigorous treatment was continued, but the symptoms notwithstanding increased, and he died on the morning of the 29th of July.

"The ball was found lodged near two inches deep in the substance of the right posterior lobe of the brain; a considerable quantity of pus surrounded it; some inflammation of the brain and its membranes was observed, but it was much less than might have been expected." 107.

In the next case, the ball did not lodge nor irritate the brain, and the result was more fortunate.

*Case.*—A. Clutterbuck, 61st regiment, aged twenty-five, was wounded in the back of the head by a musket-ball at the battle of Toulouse, on the 10th of April, 1814. He felt little inconvenience from the wound the first two days. On the 14th he complained of severe pain in the head, giddiness, and dimness of sight; the face was flushed, pulse hard and frequent. Twenty ounces of blood were taken from the arm, and the wound enlarged to expose the cranium. The upper part of the os occipitis was found fractured by the ball, and a circular portion of it, about the size of a shilling, was depressed and fractured. 15th. Pain in the head much abated; no giddiness, dimness of sight, or any unfavourable symptom; pulse still hard. V. S. ad ℥xx. To be well purged. 19th. He was bled again this day to the extent of twelve ounces, as a matter of precaution. 23rd. Continues free from any bad symptom. May 8th. The wound is now much contracted. He has been out of bed for some time past, and feels no inconvenience. A small portion of the bone still feels bare to the probe but the greater portion of the depressed piece is covered with healthy granulations. No exfoliation has taken place. May 24th. The wound is nearly healed; he is in good health and spirits, and without inconvenience. Discharged to Bordeaux.

We introduce a third case of a different complexion, as it bears upon a point of treatment.

*Case.*—"William Rogers, aged 19, of the 32nd regiment, was wounded on the 16th of June by a musket-ball, which entered at the inferior angle of the left parietal bone; it knocked him down, and for a few minutes rendered him senseless. On recovering his mental powers, which he soon did, he found that he was unable to speak, not so much (as he says himself) from the want of power to form words, as from the incapacity of giving them sound. He was conscious of everything passing around him, and reasoned correctly: he retired out of the reach of shot, and then lay down for the night. On the following morning, finding the picquets retreating, he fell back himself on Brussels, where he was examined and dressed. On the morning of the 18th he reached Antwerp on horseback, very giddy, and overwhelmed with fatigue, fasting and watching; he was admitted into the Minime General Hospital, and put to bed, when he soon fell into a sound sleep, which with some tea refreshed him much.

"June 19th. On examining the wound, the ball was found to have passed obliquely upward and backward at least two inches, and could be distinctly felt with a probe. It gave more the idea of having raised the outer table than that of having depressed the inner; both tables must however have been displaced. The defect in speech was in some measure restored, and this with giddiness were the only symptoms of compression. A poultice was placed over the wound, a sharp purgative given, and spoon diet ordered.

"20th. The pain and giddiness having increased, with annoyance from noise and exposure to light, twenty-six ounces of blood were taken from the arm. The following day the purgative was repeated, and the patient was much relieved: a faltering in the speech continued for many days. Everything went on well, the wound was nearly healed, and he was considered almost fit to be discharged, when, on the 16th of July, the wound began to open; on the 18th it was dilated and a portion of the cranium removed by the forceps, which was soon followed by symptoms of inflammation of the brain, and twenty ounce of blood were taken immediately from the arm; purgatives and diaphoretics were ordered, and the strictest abstinence enjoined. 23rd. Venesection was repeated, as well as the other means usually adopted to reduce high action. 24th. Completely relieved. Saline mixture continued; a little meat soup allowed. 26th. Another portion of the cranium removed, the dura mater being fully exposed; the general health in the best state.

"August 3rd. Doing remarkably well; the wound healthy; the pulsation of the brain evident; the power of speech perfectly restored. The ball yet remains in according to the opinion of the patient (who is a fine intelligent lad), and he thinks has gradually descended towards the petrous portion of the left temporal bone. Sent to England at the end of the month well." 106.

Mr. Guthrie observes, upon this case, that the bone and ball ought to have been removed in the first instance. The subsequent operation perilled his life, and as he was discharged with the ball lodged (though, by-the-bye, this does not seem to us very likely, the dura mater being uninjured, and no symptoms existing), it is probable that he did not long survive.

Mr. Guthrie relates a case for the purpose of showing to what extent depletion may be advantageously carried.

*Case*—Lawrence Moore, æt. 27, was knocked down on the night of the 6th of April, 1816, by a blow of a stone, which fractured the upper and left edge of the frontal bone, the depression being about an inch and a half square. V. S. ad ℥xxv. Took out the detached pieces of bone and dressed the wound simply; he lost about twenty ounces of blood during the operation. He had a pretty good night, but, on the 7th, pulse small and very hard; head feels to himself full, and gives the sensation as if it were bound with an iron hoop (his own words); eyes very suffused. V. S.

ad ℥l. with relief. At 8 P.M. the pulse having risen, V. S. ad ℥xxv. with benefit. Elect. scammon. ℈ij.

8th. Awoke better, but at 9 A.M. the pulse had risen to 130, hard and small; has a severe throbbing sensation in the head over the seat of the injury; tongue white and dry. V. S. ad ℥xl. with further benefit.

10th. Has been well purged; tongue clean, pulse more natural, eyes much depressed, the redness has left them, the fulness of head is also gone, and on the whole he is doing well. The discharge of a sanious nature, the wound externally like ochre. In the afternoon the pulse rose, but was not so hard as to indicate the use of the lancet; has pain and fulness over the injury. Was purged with senna, &c. and after this had no further symptoms of consequence. He was discharged cured on the 23d of June.

"This case," says Mr. G. "shows the advantage to be obtained by removing such fractured and depressed portions of bone as might irritate the dura mater and brain if allowed to remain, and also demonstrates the very great extent to which blood may be drawn in strong and healthy persons, in a short period (160 oz. in three days). When the symptoms were not so immediately urgent as to demand the use of the lancet, the free exhibition of drastic purgatives was attended by the best effect."* 109.

A want of method in Mr. Guthrie's writings, and an absence of logical precision in his reasoning, render it not unfrequently difficult to determine exactly what he intends, or what certain cases are meant to establish. But we gather from some remarks upon the cases, that if the compound fracture be attended with moderate depression, Mr. Guthrie would not attempt to elevate it, provided there were no urgent symptoms, whilst if he thought there were pointed pieces projecting inwards, and likely to irritate the brain, he would elevate. "If," he observes, "the examination of the depressed part had led to the apprehension that such points of bone did exist and were sticking into and irritating the dura mater or brain, I should have removed them, under the belief that although they might not at the moment have given rise to any other symptoms than those which depended on the blow, the time would come when they would scarcely fail to cause those which usually accompany the formation of matter within the skull; or if this danger should also have been avoided, that the evils which have been noticed from p. 79 to 84 as occurring at a later period, and which ultimately require the same operation for the relief of the patient after months of acute suffering, might be encountered; the cases at the pages indicated were referred to solely for the purpose of showing that, although a person might temporarily recover from an injury

* "Certain diseases give a peculiar tone to the circulatory system, enabling it *to bear*, and causing it *to require*, great loss of blood in their treatment; they are inflammations of the serous membranes and parenchymatous substance of organs. Other diseases induce this effect in a much slighter degree; such are the inflammations of the mucous membranes. Lastly, other diseases render the system unduly susceptible to the effects of loss of blood: these are the class of irritations, as gastric and intestinal disorders and irritations. Dr. Marshall Hall in the Gulstonian Lectures for 1842.

in which a portion of bone was allowed to remain an irritating substance to the brain, it did not follow that such recovery should be permanent. If there be a doubt on the mind of the surgeon whether there are or are not any portions depressed and irritating the brain or its membranes, he should wait; and in this it is that the real difference between modern surgery and that of the olden time exists with respect to adults." We are not quite sure that this is not better practice than trephining indiscriminately in every case of compound fracture with depression.

The following case supports, as far as one case can be considered to do so, this view.

*Case.*—" Captain R., aide-de-camp to General Sir L. Cole, received a wound from a musket-ball at the battle of Albuhera on the anterior and middle part of the left parietal bone at its junction with the frontal, which fractured it, causing some slight depression. He was rendered insensible at the moment, and was brought in the evening to the village of Valverde, where the insensibility was shortly followed by symptoms of inflammation, which were subdued by repeated bleedings, under which he gradually recovered, and remained well until killed at Pampeluna. The division of the scalp gave rise to no additional symptoms." 111.

Mr. Guthrie did not use the trephine, because the broken portions of bone did not on examination appear to press unequally on the dura mater, and it was presumed that the moderate degree of pressure which ensued from the depression might be borne with impunity, as it did not seem likely to be accompanied by the projection inwards of any pointed pieces which might irritate the brain. The result confirmed the supposition.

But the uncertainty that attaches to Mr. Guthrie's meaning will perhaps appear to others, as it does to us, from the passage which immediately follows the preceding. We supposed that Mr. Guthrie was treating of compound fracture with depression, that difficult and doubtful case in practice; yet the observations we shall quote seem to have reference to simple fracture.

" When a fracture is accompanied by depression, and the broken portion or portions of bone would seem to be driven into the dura mater or the brain, or to press so unequally upon them that as much mischief is likely to ensue from leaving, as from removing them, and especially in an adult or middle-aged man, less harm will in general follow from ascertaining the fact, by dividing the scalp, and removing the broken pieces, than by doing nothing, more particularly when the presence of a foreign body is ascertained. If there be no symptoms indicative of mischief below the fractured part, the surgeon must then decide, after the best estimate he is able to make of the probable evil which will occur from allowing the broken or depressed portions of bone to remain. I have already stated, page 104, that according to my experience an incision through the scalp renders the dura mater very little more liable to suppuration than it is without this; nevertheless that trifling degree of liability should not be incurred without an absolute necessity. I have now under my observation a child four years old who fell out of a window and has driven or bent in a portion of the frontal and parietal bones of the top of the head. The depression and fracture can be distinctly felt, but as there are no symptoms indicating any immediate mischief, there can be no reason for interference.

" I have said, page 102, that in young persons the brain will bear a greater degree of pressure and of irritation with impunity than it will in persons of mature age, that by far the greater number of cases in which recovery has taken place after fracture and depression of the skull with injury of the brain, and even loss of its substance, have occurred in children or in persons under the adult age; greater reliance may therefore be placed on the powers of nature in them, and less frequent recourse may be had to the aid of operative surgery in order to prevent mischief than in adults, even when the bone is fractured as well as depressed. 112.

Two cases are quoted, to be contrasted. The first was recorded in the Lancet, by Mr. Roberts, of Bangor. It was that of a little boy, in whom he allowed a large piece of bone to remain depressed and forced perpendicularly into the brain, and which appeared to him to be too firmly imbedded in it to admit of extraction. Several portions of brain were lost or removed, the child suffered from convulsions, became paralytic on the opposite side to the injury, yet gradually recovered, three pieces of bone coming away in less than ten weeks.

The other case occurred to Mr. Liston. It was that of a boy eleven years of age, who had been thrown out of a cart eleven weeks before, and had his head cut in two places by a stone bottle. The wound on the anterior superior part of the head was the most serious, and from this an angular piece of the bottle was removed. He was insensible for one week after the accident, but gradually recovered, and could walk at the end of a month. A few weeks afterwards he lost the power of speech for three days, which he recovered on a profuse discharge of matter taking place from the wound, together with vomiting. Three days after his admission into the University College Hospital, Mr. Liston examined the bone, and finding a fissure with some little depression, he applied the trephine, when two angular pieces of the inner table were found projecting much inwards on each side of the fissure, and were removed. The child did well. On these cases Mr. Guthrie remarks:

"In the first there was opening sufficient to allow of a free discharge of matter as it was secreted, and for the removal of all irregular-shaped pieces of bone. In the second the opening was not sufficient, and the irregular-shaped pieces of bone could not be removed. In the first case the trephine was unnecessary; in the second its use was imperatively called for, and it was successful." 113.

Mr. Guthrie makes a remark which appears to us a very just and not an unimportant one; it is this,—that the cases of recovery recorded bear a very deceptive proportion to the fatal cases that are not recorded. Few relate an unsuccessful one, in which either the post-mortem examination proves that something has been overlooked, or that the injury was beyond remedy by any means at present known. This makes calculations founded upon published cases so fallacious.

Some cases are given of fracture with depression and injury of the brain, in which modes of practice of a very opposite character were attended with an equally successful result. Such are the circumstances which abound in medicine, and render it difficult to say what is and what is not right to be done. General rules must be charily laid down and cautiously acted on, experience and judgment constantly stepping in to modify them. Mr. Guthrie's opinion is expressed decisively:—

"The result of my experience has rendered it imperative in my mind to remove at once all portions of bone or foreign substances which may have or may be supposed to have penetrated the dura mater in adults, although no symptoms of compression should be observed; and generally in children, whenever it can be done without difficulty, and especially when symptoms of compression are present. If the wound in the dura mater should not be sufficiently large to allow the offending body to be extracted through it, the opening must be increased to enable it to be withdrawn without further laceration; and all substances which are irritating, or are likely to irritate the brain, should be removed in the first instance, as I have already suggested, page 92, unless the attempt should be forbidden by the occurrence of convulsions, by the inability of the surgeon to seize the extraneous body, or by the evidence of the great suffering which it occasions; and all blood which may be extravasated should be carefully and lightly removed." 117.

It appears, then, that it is the probability of wound of the dura mater, that leads Mr. Guthrie to operate. But in compound fracture with depression, this probability must be a frequent one, and therefore we may presume that the operation will be a common one. In fact Mr. Guthrie's practice comes very nearly, after all, to Sir Astley Cooper's in this instance, while the former seems to advocate the use of the trephine in simple fracture with depression to an extent to which most surgeons are indisposed, perhaps, to go. But we repeat that the want of arrangement in Mr. Guthrie's observations, and the mixing up of one subject with another, render it exceedingly difficult to say what his sentiments really are.

He goes on, for instance, to state :—

"I have shown by the case of the soldier, p. 50, by that of Clayton, p. 70, of Capt. R., p. 111, and by others, that every depressed portion of bone accompanied by fracture, and especially on the back part of the head, need not necessarily be removed. When the fractured and depressed bone is accompanied by symptoms of compression in an adult, which continue after the usual antiphlogistic means and remedies have been employed in vain, and appear to increase rather than to diminish, the broken and depressed portion should be raised; for although the brain will bear and accommodate itself to pressure in many persons in a manner which could not be either foreseen or expected, it will not do so in all; and the removal of the bone offers the best chance for relief, whether the mischief has arisen from the pressure made by it, or occurs from the extravasation of blood beneath. I have on several occasions found the principal symptom of compression to be a fixed pain in the part; and although the state of the fracture and depression would not alone have rendered the removal of the bone positively necessary, I did not hesitate about removing it when this symptom was present; and I have generally seen the pain subside after the operation. The case related by Mr. S. Cooper, to which I have referred, p. 78, is most useful, from the fact which followed the removal of the bone, viz. that the patient, who was before in nearly a lifeless state, instantly sat up in bed, looked around, and spoke rationally. There was scarcely one of those great battles or seiges in the Peninsula at which I was present, where a nearly analogous case did not occur.

"The greatest discrimination is required in cases where the extent of the injury is not so manifest, and in which there is more room for doubt. In most cases in which a slight or moderate degree of fracture and depression of the skull has taken place, the symptoms of concussion are present as well as those of compression. The symptoms of concussion are however coeval with the injury; and although those of compression may take place almost instantaneously, they more usually occur at a later period of time. The symptoms of concussion may nevertheless continue for days, and more particularly the insensibility, or that state which is approaching to it, complicating the case and embarrassing the practitioner. In a child or young person the symptoms of compression or irritation, when they appear even at a secondary period, may pass away under further moderate depletion; but in an adult any undue delay in giving the necessary relief by the removal of the depressed portion of bone, will in general be destructive to the patient. It is the irritation caused by the depressed bone on the dura mater, and communicated to the brain, which gives rise to the unfavourable symptoms, and to the formation of matter which follows." 119.

This is merely reverting to matters which had been treated of and settled long before.

But to proceed. Our author arrives at secondary formations of pus.

Mr. Guthrie touches on the formation of pus within the cranium by a sort of *contre coup*.

"When a very severe blow, accompanied by a shock, as from a fall, has been received on the head, and the skull is so thick and strong as to be able to resist the violence thus offered without being broken, or is only slightly fractured, the vibration or *tremoussement* is directly communicated to the brain, giving rise to laceration or bruising of its structure in various situations, to the rupture and separation of the vessels of the dura mater from the bone to which they are attached, and to derangement of other parts, which will in all probability be followed by inflammation, and may even terminate in the formation of matter under the dura mater as well as above it, and even in the brain itself. This is said to take place by 'contre coup' when it takes place in any other part of the head than that which is struck, of which Mr. Shaw gives two cases: and of instances of which the older French authors are so profuse both in the explanation and in the fact. The cases related by Mr. Shaw are truly cases of laceration, the accompaniment and the consequence of concussion of the brain, and were not relievable by the art of surgery; but they are not exactly what the older surgeons particularly distinguished as injuries by 'contre coup,' where the blow was on one side, and a fracture took place or matter was formed in a circumscribed spot on the other, which cases did sometimes, although rarely, admit of relief by operative surgery." 120.

Mr. Guthrie, however, has not met with such cases unaccompanied by fracture. Nobody at this time of day would dream of making an exploratory crusade with the trephine.

Mr. G. observes that, as all well-informed surgeons are aware, when the periosteum covering the bone is bruised, or the bone is deprived of this membrane, it does not follow that the bone should die or exfoliate. In many instances the wound will gradually close up and heal as if no such accident had happened; and in most cases this termination will only be delayed by the separation of a scale of bone from its outer surface.

Mr. G. passes on to suppuration on the dura mater, and "Pott's puffy tumor." On this head Mr. Guthrie makes a remark which is not only, we apprehend, true, but easily accounted for. "Inflammation," he says, "of the dura mater proceeding to suppuration or the formation of matter between it and the bone, appears to have been a much more common consequence of injuries of the head in the time of Dease and Pott than at present. I have rarely seen a case of the secondary tumor they have described, and on inquiring of the surgeons of the different hospitals in London who are on the Council of the College of Surgeons, consisting of what may be called from their standing and position the *élite* of the surgery of London, I find it is almost equally unknown to them."

The fact is, that depletion and the influence of antimony and mercury are now so freely resorted to, that inflammation does not run a-head as it was let do in the days of Pott. Mr. Guthrie dwells on the frequency with which suppuration on the dura mater is accompanied by suppuration on the surface or in the substance of the brain.

He says:—

"Suppuration, or the formation of pus on the surface of the dura mater, is not, then, under the strictly antiphlogistic system of the present day, a common occurrence; and sufficient attention is not therefore paid to the evil which frequently accompanied it in former times, viz. suppuration on the surface and in the substance of the brain itself—the more usual cause of death in all these cases of fracture and depression which are left to the 'chirurgie expectante,' or that which has been too long delayed. On referring to the records of surgery from the earliest times unto the present moment, I find that the greater part of those who have died with fracture and depression of the skull, and whose cases are recorded, suffered from alteration of the structure or substance of the brain, and the formation of matter within it or upon its surface. I have seen and read of many cases of injury of the head without depression in which this termination ensued, as it might have done and has done from idiopathic inflammation without injury; but I firmly believe that it would not have taken place in a large proportion of those cases in which it occurred, if the present system of treatment had been pursued; or if the depressed bone had been raised to its level, and the irritation arising from undue or unequal pressure had been avoided. It must be admitted, however, that an internal part of the brain may receive such shock at the moment of injury, as well as an external part, that no treatment can arrest its progress towards evil, although the mischief may be delayed; and when the patient dies after four, five, or more weeks of alternate hope and of suffering, matter is found in some part of the brain where an injury was not suspected." 124.

Purulent matter may be deposited under, as on the dura mater, either in a circumscribed or in a diffused manner. The former may admit of hope—the latter scarcely can. Mr. G. touches on the incision of the dura mater, to evacuate blood or matter beneath it. He speaks favourably of the practice, which, however, is not to be lightly had recourse to. He says:—

"I have seen, on the removal of a portion of bone by the trephine, the dura mater rapidly rise up into the opening, so as to attain nearly the level of the surface of the skull, totally devoid, however, of that pulsatory motion which usually marks its healthy state; and an opening into it, under these circumstances, has allowed a quantity of purulent matter to escape, proving that the unnatural elevation of the dura mater was caused by the resiliency of the brain when the opposing pressure of the cranium was removed. I consider this tense elevation and the absence of pulsation to be positive signs of there being a fluid beneath, requiring an incision into the dura mater for its evacuation. It is a point scarcely, if at all noticed in English surgery, although much insisted upon in France. It was not in the slightest degree understood at the commencement of the war in the Peninsula, and was one of those points which particularly attracted my attention." 126.

Mr. G. relates several cases of an unsuccessful character—(there are unhappily too many of *them*). We shall mention the heads of a successful one.

Mr. Guthrie operated in a case after the battle of Toulouse. The dura mater rose up into the trephine hole, without any pulsation. He punctured it, when a considerable quantity of pus oozed out. The opening was enlarged, and the flow of matter was daily encouraged, until it gradually diminished, and ceased with the formation of granulations and the drawing in and cicatrisation of the part.

"Sir Astley Cooper entertained the opinion of Mr. Hunter, that a wound through the dura mater was particularly dangerous, in consequence of the tunica arachnoides which lines it being a serous membrane; and that, if the inflammation which ensued did not cease at the adhesive stage, by the consolidation of the surface which covered the pia mater with that which lined the dura mater, a diffused inflammation would necessarily follow, which might spread over its whole extent. This theoretical opinion is fairly deduced from the state of analogous membranes, such as the pleura and peritoneum when wounded. I do not apprehend however that practically the diffused inflammation is found to occur in cases of injury of the head, so often as it might be expected; in consequence probably of the more equal pressure that is kept up within the skull than in the chest or abdomen; but if wounding the dura mater be a danger that ought to be avoided, if possible, as one of great magnitude, the risk run by doing so cannot be put in comparison with that which accompanies the continuous irritation depending on the presence of a spiculum of bone, which has passed through the dura mater and is also irritating the brain beneath. Sir A. Cooper supposed that the danger would be diminished if the pia mater were wounded also, as the brain would project and fill the wound; but I am not satisfied of the accuracy of this opinion; and if I had opened the dura mater through error or design, I should not think I had lessened the evil by adding to it a wound of the pia mater, and perhaps also of the brain." 128.

Mr. Guthrie turns to injuries of the brain, which, he observes, are less formidable to those accustomed to military warfare, than to civilians who see them on a less frightful scale.

Gun-shot wounds of the skull are next treated of. Mr. G. recommends the external wound being in general enlarged by a simple incision, so as to show the extent of the depression or the size of the fragments. Where the bone is scarcely injured, or the periosteum only bruised, or even where the bone is deprived of this, it does not necessarily follow that it should die, or even exfoliate. In many instances, the wound will gradually close in and heal, as if no such evil had occurred; and in those which do not terminate so favourably, the cure will only be delayed by the exfoliation of a layer or scale of bone from its outer surface, unless the mischief should have penetrated deeper, affecting the whole substance of the bone or even the parts beneath.

"A musket-ball," continues Mr. G. "striking directly against a bone sometimes makes a hole not larger than itself with or without any radiating fracture; and one trephine, if properly applied, will often embrace the whole of the mischief, and admit of the removal of the broken pieces. The trephine should be of a large size, and as a centre pin cannot be used, it may be made to turn very well in most cases in a flat but thick bar of iron, having a hole in the middle of such size only as will admit the outside of the polished trephine to turn in it. Sufficient support for the instrument will be obtained by this means until it has made a groove in the bone for itself, when the operation may be continued as it would be in an ordinary case after the removal of the centre pin. Botal and Percy both allude to contrivances of this kind as eminently useful, and I have myself found it very advantageous.

"When a musket-ball ranges along the side or top of the head, it may break the outer and depress and fracture the inner table to a considerable extent, for the space even of three or more inches, of which the case related, page 105, is an example. I have almost always removed the broken portions of bone by means of good forceps and a straight saw, and have perhaps been as often successful as the reverse. I can see no reason for delaying the operation unless the case be doubtful, when it may be as well to wait for symptoms, as in the case above noticed. It sometimes although rarely occurs that a ball sticks so firmly in the bone that it cannot be extracted by working round it in any ordinary way, with a pointed instrument. The difficulty usually arises from the ball having half buried itself in the diploe, and so little of it being exposed, as not to admit of a firm hold being taken of it. The large trephine, used in the way I have just pointed out, has enabled me several times to overcome the difficulty. I have even found the removal of the outer table to be sufficient where the inner one has not been driven into the dura mater; but where any doubt is entertained on this point the two should be removed." 131.

A ball, or other foreign substance, may penetrate the brain directly or obliquely. When directly, it can seldom be removed, and the patient rarely survives beyond two or three days. Mr. G. has never had under his own care a case which did well after the removal of a ball, which had been deeply driven into the *anterior* part of the brain, though he has seen and mentioned several instances of recovery, where the injury had occurred towards the back part of the head, and the ball had been allowed to remain. He thinks it " better in all such cases to allow the ball to remain unmolested, which it will often do for many days, until circumstances render it necessary to endeavour to find it. When it can be felt immediately under the surface, it ought to be removed as a foreign substance, provided this can be done with little apparent inconvenience.

Passing over some cases, we find Mr. Guthrie stating that when a ball strikes the head obliquely, it may enter and pass out, or lodge. Most of these patients die. " When the entrance and exit of the ball are obvious and not far distant from each other, the splinters of bone should be removed; and if the little bridge between the openings should be injured, the whole should be taken away by the straight saw; an operation which cannot however be necessary in the first instance, if the portion of bone should be apparently sound."

Perhaps the best case referred to is that of Baron Larrey, though it is probably too favourable a sample to be an ordinary one.

*Case.*—" A soldier of the 18th demi-brigade was wounded during the first revolt at Cairo by a musket-ball, which pierced the middle of the frontal bone near the longitudinal sinus, without injuring the dura mater, and passed backward between it and the bone as far as the occipital suture. The accident was followed by the usual symptoms of compression, the soldier, however, always complaining of pain at the back part of the head at a spot opposite to the entrance of the ball. I introduced a gum-elastic sound through the hole in the frontal bone along the track which the ball had made, until I discovered it by the resistance it offered to the further passage of the sound, and by the inequalities of its surface. Having thus ascertained the distance at which it was situated, I applied a large trephine immediately over the part by measurement; a quantity of pus was immediately evacuated, and I easily extracted the ball, which was depressing the dura mater and brain. The man after this recovered." 133.

A case, in some degree, similar, occurred to Mr. Guthrie, but the brain was injured in front, and the man died.

After the battle of Toulouse Mr. G. had a case in which the ball went through the bone and brain, and lodged under the scalp, which it could not penetrate. The man died.

A small ball is sometimes so flattened against the skull, as to escape detection. A soldier was wounded by a ball on the side of the head, which was not supposed to have lodged. The wound did not heal, a small opening remaining, although no exfoliation took place, and the bone did not seem to be bare. On dividing the scalp, Mr. G. found a small ball quite flat, which had sunk down a little below the hole left for the discharge it had occasioned.

When a larger ball or a piece of a shell strikes the head, the fracture is usually extensive, and portions of bone, or a piece of the shell itself, are often lodged in the substance of the brain. These cases are generally unfortunate.

A fall, particularly on the vertex, may, it is well known, separate the sutures, usually a fatal case. But "a suture may be separated by a musket-ball, which impinges with a moderate degree of force directly upon it, with less danger. It can only however happen in young persons in whom the sutures are not obliterated as they are in elderly ones, and in general takes place when the ball happens to lodge as it were between the bones concerned in the formation of the suture."

*Case.*—A heavy dragoon was wounded at the battle of Salamanca by a musket-ball in the body, which caused him to fall from his horse, and injured the top of his head. Little attention was paid to him until mischief was suspected from the lethargic state into which he fell, and which could only be attributed to the blow on the head, where a tumor was observable. This, on being divided, showed a separation of the edges of the sagittal suture, from which some blood flowed. Two crowns of the trephine were applied on the twelfth day, in order to admit of the free discharge of some blood which had been extravasated from a wound in the longitudinal sinus, after which the symptoms subsided, and the patient gradually recovered.

Mr. Guthrie has, in four instances, seen irremediable blindness occur in the following manner. A ball passes through the fore-part of the head from side to side, but it does not injure the brain, coursing immediately below it and through the back part of both orbits.

We have some cases of injury of the frontal sinus and remarks upon them.

"The danger of injuries to the frontal sinuses has been greatly exaggerated, and vanishes in a great degree, when attention is paid to their structure. The uncertainty of the depth of the cavity between the tables of the bone and the irregularity of the exposed surface of the inner table, which may through carelessness be mistaken for depression, should be remembered. Larrey relates the history of two cases of fracture from musket-balls which he treated with success in the campaign in Egypt and Syria without leaving any aerial fistula; by the application of a large crown of the trephine on the exterior table, so as to expose the inside of the frontal sinus, when a smaller instrument was readily applied, so as to enable him to raise the depressed or broken portions of the inner table; a practice which ought to be imitated in all such cases which require the operation of the trephine." 136.

*Case.*—A soldier was wounded by a ball, which struck him on the lower part of the right side of the forehead, fracturing the external wall of the frontal sinus. On examination, the ball could be felt lodged in the sinus, from whence it was readily removed by enlarging the opening, and the man recovered without any bad symptoms. Le Dran gives a case in which a ball having entered in this way, was found a year afterwards lodged in the brain by the side of the sella turcica.

Mr. Guthrie has never seen a case in which, after wound of the frontal sinus, the air did more than raise the cicatrix, though he has often had difficulty in closing the external opening.

Mr. Guthrie relates some cases of injury of the brain, by foreign bodies which reached it through the orbit. Perhaps the following is as good an instance of the insidious and dangerous nature of these accidents as any.

*Case.*—" A boy, nine years of age, was brought to the Ophthalmic Hospital struck by his playfellow with the end of a thick iron wire on the right eye, which blackened it. There was no external wound; but as there was some bloody chemosis at the upper part and inside, there was a probability of the wire having penetrated deeply, although the opening could not be discovered by the probe. The accident had happened two days before, and the boy had vomited shortly afterwards, and had eaten little since, although he did not think himself ill. He was well purged, and cold water was desired to be applied externally. Two days after he returned, complaining of sickness, headache, and some pain over the brow, and looked ill. It was now suspected that the instrument had penetrated into the brain, although the ecchymosis was in a great measure gone and the eye was unaffected. He was bled freely from the temple of that side by leeches, and calomel and jalap were given him so as to act fully. He did not attend the next or fifth day, but on the sixth his mother came to say he had been very ill, and delirious and restless all night. On going to visit him, he was found stupefied, answering with difficulty and incoherently; pulse very quick, skin hot and dry, with some convulsive twitches of the face and arms; pupils slightly obeying the influence of a strong light, but not dilated. He was again bled freely from the temple, but his breathing became more difficult, he fell into a comatose state, and died in the night. On examining the head, the stiff iron wire was found to have passed under the upper eyelid between it and the eye, through the posterior part of the orbitar plate of the frontal bone and into the anterior lobe of the brain, which was softened at that part, and bedewed with a little matter." 137.

Cases of this description, and they are not *very* rare, are calculated to inspire caution. It would have been well not to have suffered this boy to attend as an out patient. Had he been more closely watched, his chance might have been better. An injury of the longitudinal or lateral sinuses, which allows the blood to escape freely, is accompanied with little danger. But it is very fatal when the blood is permitted to accumulate.

Mr. G. makes some remarks on what is called fungus cerebri. It is of two kinds, and occurs at different periods of time. The first kind is principally composed of coagulated blood, usually appears immediately after, or within two days after the injury, and is generally fatal. The second takes place at a later period, and is formed for the most part of brain. They seldom occur either where the loss of skull has been great, or where, with a small opening, the dura mater is uninjured.

In the first kind of protrusion the dura mater must necessarily be torn to some extent, and the tumor which comes through it is of a dark brown colour, glazed and covered in general by the pia mater. These protrusions were accompanied, in the cases that Mr. G. has seen, by symptoms of inflammation of the brain and its membranes, coma not occurring till near the fatal termination. He has seen the protrusions torn off, and was able to satisfy himself "that they all arose from hæmorrhage into the substance of the brain, probably immediately below its surface, which became augmented in size as the inflammation proceeded, and was gradually protruded at the part where there was the least opposition. When the tumor was torn off little hæmorrhage ensued, but a dark brown bloody cavity was seen in the substance of the brain; or when cut off and examined, the protruded part seemed to be covered by the pia mater, with or without a layer of cerebral matter, and was made up generally of coagulated blood." Mr. G. never saw a case recover. He feels disposed to recommend that "all such bloody tumors should be cut off on a line with the surface of the skull as soon as they appear above it, or that they be removed altogether, so as to allow of a free discharge of blood or of any fluid which may be collected under the dura mater. Blood cannot be drawn under these circumstances in any other way so well as from the surface or the substance of the brain itself, and a free discharge for any matters which may be collected beneath the bone is essential to the safety of the patient." The general treatment should be that of inflammation of the brain, of which this must be looked on as a symptom.

In the second kind of protrusion, which occurs when the active inflammatory symptoms are declining, Mr. G. is convinced that the tumor is formed by the substance of the brain, though he is *not* convinced that the loss of brain is invariably proportionate to the extent of the protrusion. He thinks that as the precise quantity which a person may lose with impunity has not been ascertained, it may be as well to deprive a patient of none, provided its removal can be dispensed with. In one of some cases the nitrate of silver was lightly used—moderate pressure is the remedy advised by Mr. G. The pressure, he states, was graduated, according to the feeling of the individuals; when made too firmly it gave rise to swimmings and pain in the head, retardation of the pulse, a sense of sickness and fainting, and even in one instance to syncope. Pressure could only be borne when very lightly applied whilst the protrusion was increasing, but could be gradually augmented when it became stationary, and during its diminution and secession. The pressure was continued until after the wound had healed. Mr. Guthrie observes:—

"The preceding cases prove that persons may recover after having had a protrusion of the brain, without, as well as with the loss of a portion of its substance, the difference in all probability between the cases being dependent on the degree of mischief which gave rise to them. In all those which I had an opportunity of examining after death, and the injury in all was on the top or upper part of the sides and back of the head, the protrusion was manifestly a part of the substance of the brain, and firmer than the hemisphere beneath, which was soft, pulpy, and of a yellow and sometimes of a reddish colour, the lateral ventricle being filled with a sero-purulent matter, pus itself being spread over the surface and intermingled with the pulpy structure, into which the brain had been changed. That the protrusion was the consequence of low inflammation of the brain, there could be no doubt; and that greater caution had been necessary during the progress of the mischief than had been enforced, was in all probability the fact. It was the observation of this and of other circumstances not less important which led me to enjoin that rigid system of management which I have insisted upon in all cases of injury of the head. There can be no doubt of the formation of many of these protrusions being aided by the opening which has been made in the dura mater, which would have restrained their growth if it had been sound. The dura mater should never therefore be opened if it can be avoided, and the protrusions thus formed are the most likely to be withdrawn as the irritation which gave rise to them subsides." 144.

Mr. Guthrie, like most modern surgeons, is averse to excision. There are some remarks on abscess of the liver, consecutive to injury of the head—an affection which probably only follows such injury as it may any other.

"When a person has received a serious blow on the head, which has given rise to an exfoliation of the bone, or to a very slight depression of the skull, he is rarely restored to his previous healthy and natural state. The scalp adheres firmly to the bone beneath instead of sliding loosely over it, and a deep hollow is formed, which would imply that greater mischief had been done, and a greater loss of bone had been sustained than actually took place; and this is the more remarkable when pieces of bone have been removed. I have now under my care, for disease in other parts, Major D. of the Indian Army, who was wounded on the left side of the forehead at its upper part by a musket-ball at the assault of Mahidpoore. Several pieces of bone were removed, and the pulsation of the brain was evident in the discharge. I can push the point of my little finger into the hole left by the cicatrisation of the wound to an extent I should not have suspected if I had not been aware of the fact. This officer suffers from head-aches, augmented or brought on by any exertion of body or mind. He cannot bear exposure to the heat of the sun. He can scarcely drink three glasses of wine without feeling its effect. In all these cases, and I could relate many, of persons of education, they can bear no great exertion of any kind. They fall down under exposure to heat. They are easily inebriated, rendered furious by a small quantity of liquor, and often become stupefied, comatose, or even die suddenly. In addition to these evils, which may be avoided by care, many are subjected to fits, which are apparently epileptic; and others suffer from such intolerable pain in the part injured, as well as in the head generally, as to be rendered miserable and desirous of seeking relief at any risk.

"These injuries are often accompanied during their progress by mental defects which time does not always remove. The memory is very often much impaired. It is frequently defective as to things as well as to persons. The sight of one or both eyes may be impaired, or even lost. Ptosis, or a falling of the upper lid, is not an uncommon although a more curable defect. Speech is not only difficult, but the power of uttering certain words is often lost; a language is occasionally for a time forgotten, and a sort of conventional one has even been adopted, in the manner mentioned by Sir A. Cooper, the Baron Larrey, Sir B. Brodie, and in the case related by Dr. Hennen, which was under my own observation. The more serious evils which befall these unfortunate sufferers are aberrations of mind, rendering some degree of restraint necessary, or a state of fatuity, which is not less distressing. These intellectual defects are often accompanied by various states of lameness and debility, from which there is but little hope of recovery." 150.

We have now presented a very full account of this interesting volume. The practical surgeon will find it of great value, and reference will often be made to its facts. We trust that a long career of usefulness is still open to its author.

